# Longitudinal change of reticular pseudodrusen area in ultrawide-field imaging

**DOI:** 10.1038/s41598-022-25947-z

**Published:** 2022-12-26

**Authors:** Je Moon Yoon, Young Joo Choi, Don-Il Ham

**Affiliations:** Department of Ophthalmology, Samsung Medical Center, Sungkyunkwan University School of Medicine, 81 Irwon-Ro, Gangnam-Gu, Seoul, 06351 Korea

**Keywords:** Retinal diseases, Macular degeneration

## Abstract

This study aimed to investigate the longitudinal change in the reticular pseudodrusen (RPD) area in the fundus and its association with late age-related macular degeneration (AMD). 91 RPD eyes (55 patients; age 67.9 ± 7.3 years) with > 5 years’ follow-up (6.8 ± 0.9 years) from a single medical center were enrolled. Ultrawide-field photography images were analyzed using the concentric rings method, and the RPD area was semi-quantitatively classified according to the affected segment number into central, intermediate, and extensive types. Correlations of longitudinal changes in the RPD area and late AMD risk were investigated. RPD area increased significantly during the follow-up (p < 0.001). The increase rate correlated with age (r = 0.207; p = 0*.*048), RPD area at first visit (r = − 0.222; p = 0*.*035), and the decrease rate of subfoveal choroidal thickness (SFCT) (r = 0.217; p = 0*.*039). Many central (18/49, 36.7%) and intermediate (15/23, 65.2%) types switched to the more advanced type during the follow-up. Macular neovascularization and geographic atrophy developed in 12.3% and 18.7% of patients by 7 years. Late AMD incidence was significantly higher in eyes with large than in those with small RPD areas (p = 0*.*002). Larger RPD area at baseline, faster increase in RPD area, thinner SFCT, rapid decrease in SFCT, and the presence of late AMD on fellow eye were associated with late AMD. All RPD areas progressively increase over time. The regular assessment of RPD area may help to predict late AMD risk in RPD eyes.

## Introduction

Reticular pseudodrusen (RPD), also known as subretinal drusenoid deposit (SDD), accumulates in the subretinal space above the retinal pigment epithelium. RPD is an independent risk factor for late AMD in eyes with non-late AMD, if the fellow eye has MNV as shown previous studies^1,2^. Recently, a post hoc analysis study of AREDS (age-related macular degeneration study) and AREDS2 reported that RPD represent a risk factor for progression to late AMD alongside soft drusen and macular pigmentary abnormalities^3^. RPD is particularly associated with geographic atrophy (GA) and type 3 neovascularization^4–8^.

To understand the prognosis of eyes with RPD, it is necessary to understand the evolutionary changes in RPD over time. It has been reported that RPDs are dynamic lesions with spatiotemporal changes^9–12^. In OCT studies, RPD lesions showed progressive increases in height and then regressed with outer retinal atrophic changes over time^13^. In photographic image analysis, RPD usually starts at the superior macula, near the superior temporal vessels, spreading upward and sometimes outward to the mid-periphery or far-periphery^4,6,8,11,14^. RPD may also occur nasal to the disc, and sometimes fade out after the onset of macular neovascularization (MNV)^11,15^. Moreover, RPD regresses when outer retinal atrophy (ORA) or complete RPE and outer retinal atrophy (cRORA) develop^11^. It is noteworthy that the fundus distribution of RPD varies from the localized form to the diffuse form, and that the diffuse form of RPD is associated with the highest prevalence rate of late AMD^8^. Moreover, initial location outside of the macular area and diffuse distribution of RPD were shown to increase the risk for late AMD development^16,17^. Therefore, longitudinal changes in the RPD area over the whole fundus should be investigated for the prediction of disease progression. Several previous studies investigating RPD in the central area have reported increased or decreased RPD areas^9–13^. However, although it has been reported that the RPD area outside the posterior pole undergoes changes, no previous longitudinal study has reported its measurements^11^.

This study sought to investigate the long-term changes in RPD area from the posterior pole to the periphery, using a semi-quantitative ultrawide-field (UWF) imaging method, as understanding these changes over time will be useful in predicting the clinical course and risk of AMD in eyes with RPD.

## Results

Of the 284 eyes from 142 patients that were reviewed, 91 eyes from 55 patients met the inclusion criteria. The demographic and clinical features of the patients are summarized in Table 1. The mean age at first visit was 67.9 ± 7.3 years (median, 69 years; range, 50 to 85 years). All patients were Korean, and 51 patients were women (92.7%). The mean duration of follow-up was 6.8 ± 0.9 years (median, 6.8 years; range, 5.1‒8.4 years). At the first visit, 67 eyes (73.6%) had soft drusen, and 7 eyes (7.7%) had late AMD in the fellow eye, 5 of which had soft drusen. Large drusen and pigmentary changes were observed in 28 eyes (30.8%) and 38 eyes (41.8%), respectively.

**Table 1:** Demographic and clinical features of the entire cohort.

Feature	Data
No. of eyes	91
No. of patients	55
Women	51 (92.7)
Laterality (OD:OS)	46:45
Age at first visit (years)	67.9 ± 7.3
Duration of follow-up (years)	6.8 ± 0.9
BCVA at first visit (logMAR)	0.06 ± 0.08
Spherical equivalent (diopters)	− 0.13 ± 1.25
CRT at first visit (μm)	267.2 ± 32.9
SFCT at first visit (μm)	170.4 ± 57.9
**Ocular comorbidities**	
Pseudophakia	31 (34.1)
Soft drusen	67 (73.6)
Epiretinal membrane	4 (4.4)
Vitelliform lesion	2 (2.2)
**Medical history**	
Diabetis mellitus	9 (16.4)
Hypertension	26 (47.3)

Data are shown as mean ± standard deviation or number (percentage) unless otherwise indicated.

*BCVA* best-corrected visual acuity, *CRT* central retinal thickness, *logMAR* logarithm of the minimum angle of resolution, *SFCT* subfoveal choroidal thickness.

### Longitudinal changes during the follow-up period

A comparison of the clinical characteristics of patients between the first and final visits is summarized in Table 2. The final mean best-corrected visual acuity (BCVA), central retinal thickness (CRT), and subfoveal choroidal thickness (SFCT) were significantly decreased as compared to the values at the first visit (p < 0*.*001). The mean rate of decrease in CRT and SFCT was 5.04 ± 9.15 and 4.44 ± 3.55 μm/year, respectively.

**Table 2:** Comparison of characteristics between the first visit and the final visit.

Characteristic	At the first visit (N = 91)	At the final visit (N = 91)	p value*
BCVA (logMAR)	0.06 ± 0.08	0.15 ± 0.24	< 0.001
CRT (μm)	267.2 ± 32.9	233.1 ± 60.2	< 0.001
Rate of decrease in CRT (μm/year)	NA	5.04 ± 9.15	
SFCT (μm)	170.4 ± 57.9	140.3 ± 51.8	< 0.001
Rate of decrease in SFCT (μm/year)	NA	4.44 ± 3.55	
RPD area (number of affected segments)	21.5 ± 11.7	30.4 ± 11.9	< 0.001
Rate of increase in RPD area (segment/year)	NA	1.34 ± 0.78	
**Increase of RPD area (Total)**	NA	91 (100.0)	
ST	NA	77 (84.6)	
SN	NA	72 (79.1)	
IT	NA	77 (84.6)	
IN	NA	77 (84.6)	
**RPD in the Macula**			
ST	91 (100)	91 (100)	
SN	91 (100)	91 (100)	
IT	82 (90.1)	91 (100)	< 0.001
IN	80 (87.9)	91 (100)	< 0.001
**Type**			< 0.001
Central	49 (53.8)	31 (34.1)	
Intermediate	23 (25.3)	21 (23.1)	
Extensive	19 (20.9)	39 (42.9)	
MNV	0 (0.0)	11 (12.1)	< 0.001
Geographic atrophy	0 (0.0)	16 (17.6)	< 0.001

Data are shown as mean ± standard deviation or number (percentage) unless otherwise indicated.

*BCVA* best-corrected visual acuity, *CRT* central retinal thickness, *logMAR* logarithm of the minimum angle of resolution, *MNV* macular neovascularization, *RPD* reticular pseudodrusen, *SFCT* subfoveal choroidal thickness.

*Generalized Estimating Equation method adjusted by interval year.

**Figure 1:**
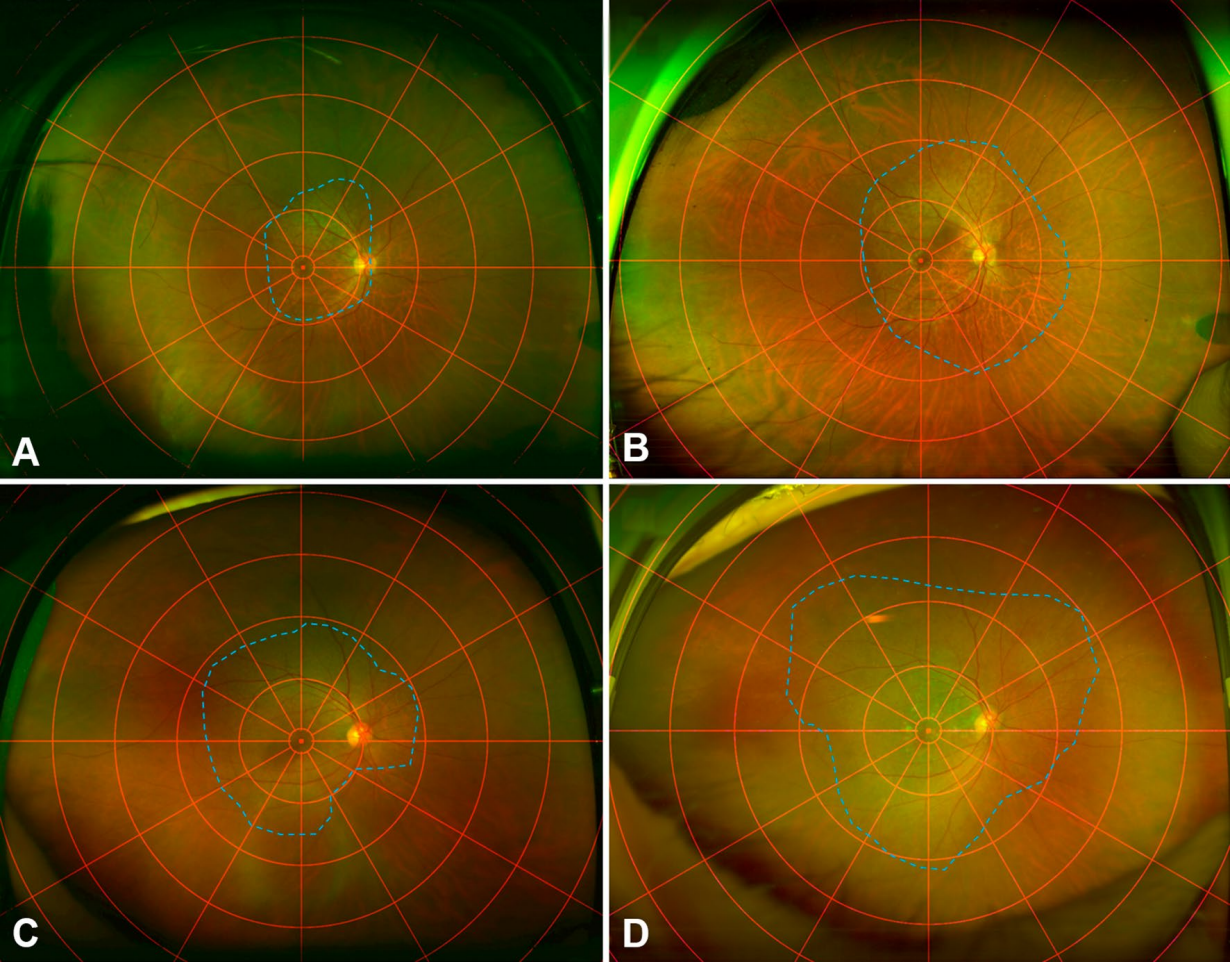
Representative cases. All images were shown with the concentric rings (orange line) and RPD area (blue dotted line). Ultrawide-field (UWF) photographs of the eye of a 59-year-old man with reticular pseudodrusen showing changes between (**A**) the first visit and (**B**) the final visit. During the intervening 73 months, the number of involved segment increased from 18 to 29 and the distributional type changed from the central type to the intermediate type. UWF photographs of a 75-year-old woman showing changes between (**C**) the first visit and (**D**) the final visit. During the intervening 99 months, the RPD area increased from 23 to 33 segments and the distributional type changed from the central type to the extensive type.

All RPD eyes showed an increase in the RPD area. The mean RPD area (the number of affected segments) at the first visit was 21.5 ± 11.7 segments (median, 19; range, 3–54). The mean RPD area significantly increased to 30.4 ± 11.9 segments (median, 28; range, 12‒65, p < 0*.*001) at the final visit. Representative cases are shown in Fig. 1.

A comparison of the RPD area changes according to the retinal area during the follow-up period is shown in Table 3. The mean RPD area in the superior half and the inferior half area at the first visit was 12.3 ± 5.8 segments and 9.1 ± 6.2 segments, respectively (p < 0*.*001). At the final visit, the mean RPD area in the superior half and inferior half area was 16.3 ± 5.6 segments and 14.1 ± 6.5 segments, respectively (p < 0*.*001). The change in RPD area during the follow-up was greater in the inferior half area than in the superior half area (4.6 ± 2.9 segments and 3.7 ± 2.4 segments, respectively; p = 0*.*004).

**Table 3:** Comparison of the area of reticular pseudodrusen according to the retinal area between the first visit and the final visit.

Retinal area	Affected segments at the first visit	Affected segments at the final visit	p value	ΔAffected segments
Superior half	12.3 ± 5.8	16.3 ± 5.6	< 0.001*	3.7 ± 2.4
Inferior half	9.1 ± 6.2	14.1 ± 6.5	< 0.001*	4.6 ± 2.9
	< 0.001*	< 0.001*		0.004^†^
Superotemporal	6.0 ± 3.0	8.0 ± 3.1	< 0.001*	2.0 ± 1.3
Superonasal	6.3 ± 3.0	8.3 ± 2.7	< 0.001*	2.0 ± 1.5
Inferotemporal	4.6 ± 3.2	7.1 ± 3.4	< 0.001*	2.6 ± 1.8
Inferonasal	4.5 ± 3.1	6.9 ± 3.3	< 0.001*	2.4 ± 1.8
	< 0.001^‡^	0.007^‡^		0.041^‡^

Data are shown as mean ± standard deviation unless otherwise indicated.

*Wilcoxon signed ranks test.

^†^Paired *t* test.

^‡^One-way analysis of variance.

The amount of increase in the RPD area correlated with the patient’s age at the first visit (r = 0.256, p = 0*.*014), and the amount (r = 0.228 and p = 0*.*030) and the rate of decrease in SFCT (r = 0.227 and p = 0*.*030). The rate of increase in the RPD area correlated with age at the first visit (r = 0.207 and p = 0*.*048), RPD area at the first visit (r = − 0.222 and p = 0*.*035), and the rate of decrease in the SFCT (r = 0.217 and p = 0*.*039). The amount and the rate of decrease in the CRT did not correlate with all parameters of the RPD area.

**Figure 2:**
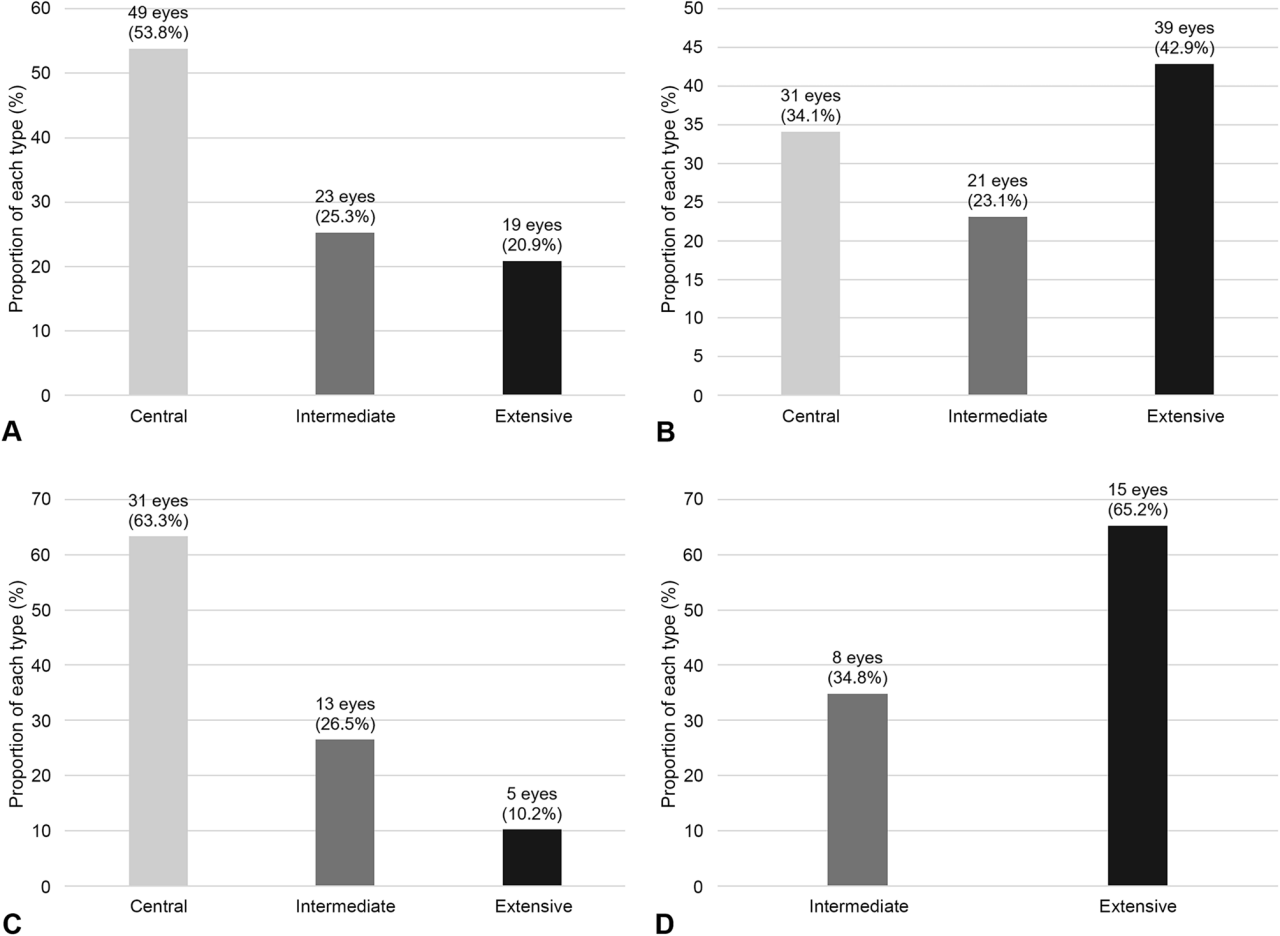
The proportion of the distributional type of reticular pseudodrusen (RPD). The proportion of distributional type at (**A**) the first visit and (**B**) the final visit. (**C**) The proportion of distributional type at the final visit in RPD eyes showing the central type at the first visit. (**D**) The proportion of distributional type at the final visit in RPD eyes showing the intermediate type at the first visit. All eyes with the extensive type at the first visit showed the extensive type at the final visit.

The distribution type changed significantly during the follow-up period (p < 0*.*001). Of the 49 eyes with the central type, 18 (36.7%) switched type: 13 eyes (26.5%) changed to the intermediate type and 5 eyes (10.2%) to the extensive type. Of the 23 eyes with the intermediate type, 15 (65.2%) changed to the extensive type. The change in the distribution profiles RPD types are shown in Fig. 2.

The intergrader agreement for RPD area measurement by the concentric rings method at the first visit was 0.996 (95% CI 0.991‒0.998, p < 0*.*001) and 0.988 (95% CI 0.979‒0.993, p < 0*.*001) at the final visit. The intergrader agreement for the type of distribution in the RPD were 0.982 (p < 0*.*001) at the first visit and 0.966 (p < 0*.*001) at the final visit.

### Incidence and risk factors of late age-related macular degeneration

**Figure 3:**
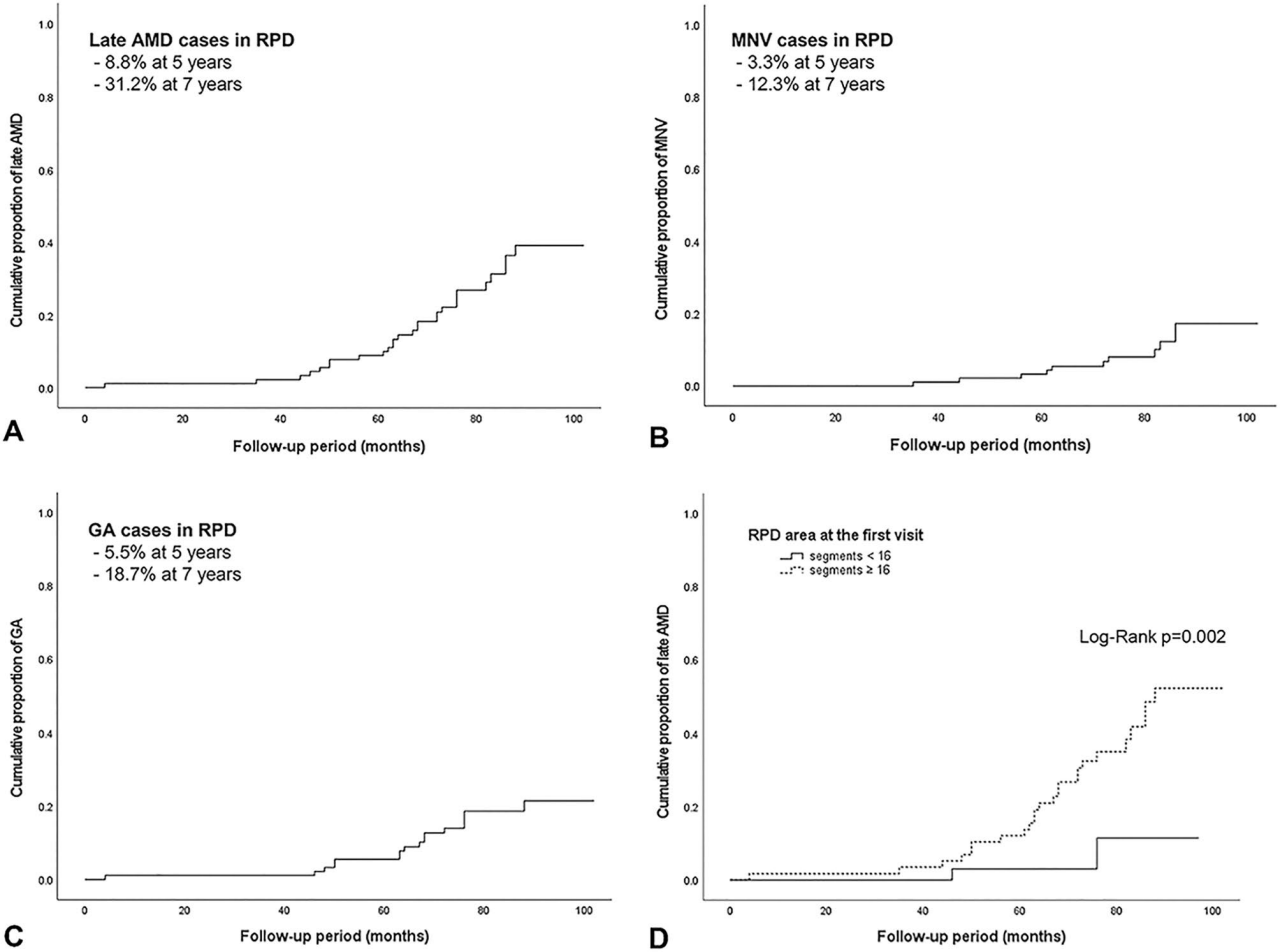
Kaplan‒Meier survival curve. (**A**) Kaplan‒Meier survival curve shows late age-related macular degeneration (AMD) in 8.8% at 5 years and 31.2% at 7 years in reticular pseudodrusen (RPD) eyes. (**B**) The cumulative proportion of macular neovascularization was 3.3% at 5 years and 12.3% at 7 years. (**C**) The cumulative proportion of geographic atrophy was 5.5% at 5 years and 18.7% at 7 years. (**D**) Eyes with RPD area ≥ 16 segments showed a significantly higher cumulative incidence of late AMD than those exhibited by eyes with RPD area < 16 segments.

During the follow-up period, MNV and GA developed in 11 eyes (12.1%) and 16 eyes (17.6%), respectively. Among the 11 eyes with MNV, 10 eyes had type 3 MNV, and 1 eye had polypoidal choroidal vasculopathy. At the last visit, 20 eyes of 67 eyes (29.9%) with soft drusen and 6 eyes of 24 eyes (25%) without soft drusen had a progression to late AMD. The Kaplan‒Meier survival curve showed late AMD development in 31.2% of patients by 7 years (Fig. 3). The cumulative proportions of MNV and GA were 12.3% and 18.7% at 7 years. A higher cumulative probability of late AMD was observed in those with larger RPD areas (≥ 16 segments) than in those with smaller RPD areas (< 16 segments) (p = 0*.*002). In Cox proportional hazard models using univariate analysis, thinner SFCT, rapid decrease in SFCT, larger RPD area at baseline, rapid increase in RPD area, and the presence of late AMD on fellow eye were found to be significantly associated with late AMD in RPD eyes. Risk factors that were statistically significant in univariate analysis and previously reported risk factors (large drusen and pigmentary changes) were included in multivariate analysis. Because the rate of increase in RPD area almost reached statistical significance, it was also included in the multivariate analysis to investigate the effect on late AMD after the other confounders were adjusted. In multivariate analysis, thinner SFCT, rapid decrease in SFCT, larger RPD area at baseline, rapid increase in RPD area, and the presence of late AMD on fellow eye were associated with late AMD development (Table 4).

**Table 4:** Cox proportional hazard model for prediction of late age-related macular degeneration in eyes with reticular pseudodrusen.

	Univariate analysis			Multivariate analysis		
	HR	95% CI	p value	HR	95% CI	p value
Age at the first visit (years)	1.032	1.121‒1.561	0.355			
Gender	12.361	0.447‒41.876	0.138	12.910	0.669‒49.093	0.090
SFCT (μm)	0.983	0.972‒0.994	0.003	0.983	0.972‒0.993	0.002
Rate of decrease in SFCT (μm/year)	1.526	1.252‒1.861	< 0.001	1.523	1.254‒1.849	< 0.001
RPD area at the first visit (number of affected segments)	1.044	1.006‒1.084	0.023	1.036	1.005‒1.069	0.023
Rate of Increase in RPD area (segment/year)	1.006	1.000‒1.011	0.056	1.006	1.001‒1.012	0.026
Large drusen	1.224	0.443‒3.382	0.697			
Pigmentary changes	1.197	0.502‒2.853	0.685			
Late AMD on fellow eye	10.801	2.578‒45.253	0.001	13.103	3.387‒50.690	< 0.001

*BCVA* best-corrected visual acuity, *CI* confidence interval, *CRT* central retinal thickness, *HR* hazard ratio, *logMAR* logarithm of the minimum angle of resolution, *RPD* reticular pseudodrusen, *SFCT* subfoveal choroidal thickness.

Significant values with p < 0.05 are indicated by asterisk.

## Discussion

Understanding the longitudinal and evolutionary changes in RPD may facilitate an understanding of the pathogenesis and prognosis of RPD. In this study, we showed longitudinal and evolutionary changes in RPD with regard to the affected fundus area. Although several studies have reported longitudinal RPD area changes in the macular area, there were no previous studies regarding the longitudinal RPD area changes outside the macula using the UWF imaging method, based on our literature search in PubMed. Additionally, this study investigated evolutionary changes in RPD without the influence of preexisting late AMD. RPD frequently disappears after the development of MNV or regresses with the development of GA^11,13,15^. Therefore, the study subjects were confined to RPD patients without late AMD at baseline, to exclude any antecedent influence of late AMD on the RPD area changes.

All eyes in this study showed a significant increase in the distributional area during the follow-up period. None of the RPD areas were static; all patients showed progressive increase toward the periphery over time. Although only the macular area was investigated, previous longitudinal studies investigating the central RPD area of non-exudative AMD eyes showed increased or decreased areas. Steinberg et al. in their study using confocal scanning laser ophthalmoscopy and SD-OCT, reported that RPD involvement was higher at the follow-up than at the baseline in eyes with early and intermediate AMD, supporting the results of the present study^10^. However, unlike the results of the present study, Kaszubski et al. reported that the mean RPD area did not change on ICGA or NIR images, and even decreased significantly on AF images among eyes that did not progress to late AMD^9^. They also reported that either regression or expansion of the RPD area could be seen. It is noteworthy that they used different multimodal imaging methods and did not investigate the RPD area outside the central area. Further, the semi-quantitative UWF imaging analysis used in this study is not appropriate for assessing small or subtle area changes, similar to those reported in previous central area analysis. RPDs are assumed to be present in the segments if any RPD lesions were found in that segment, regardless of the actual size of the affected area. Therefore, regional increase (progression) and regional decrease (regression) in the segment area could not be accurately assessed. Furthermore, the resolution of UWF imaging is lower than that of conventional photography. Thus, there might have been some regression of RPD in some regions of the central area, particularly in regions with old RPD lesions. However, the results of this study indicated that the total RPD area increased over time, if the entire fundus area is visible on imaging.

It has been reported that RPD usually first develops in the area between the upper edge of the fovea and the superior temporal vessels, and then slowly spreads, mainly in an upward direction to the midperiphery^11^. It was partly confirmed in this study that RPD starts at the superior macula area, and our results indicated that RPD can increase in all directions. Moreover, this increase was greater in the inferior than in the superior half. However, it is noteworthy that this study could not accurately assess the actual directional area increase or the directional tendency of the RPD area increase, because the concentric ring was centered at the fovea, which is not the starting point of RPD development, and because a semi-quantitative assessment was used. We consider that the RPD area increase appeared to be greater because the concentric ring was centered at the fovea and there were more unaffected segments in the inferior half at the first visit. Nevertheless, this study showed that the RPD area increases not only in the superior direction, but also in all other directions. Further longitudinal studies using quantitative area measurement methods centered at the superior macula, where RPD starts, are needed to assess the RPD area spread and rate in different directions accurately.

In this study, late AMD occurred in 8.8% and 31.2% of patients at 5 and 7 years, respectively. Compared with the previously reported incidence of late AMD in Korean RPD eyes (15.6% at 3 years)^17^, the current data showed a lower incidence of late AMD. We speculated that the small number of study subjects and selection bias were the possible reasons for the difference. The presence of early AMD with RPD, a thin choroid, diffuse distribution of RPD, and the presence of late AMD on fellow eye at baseline were significant risk factors for developing late AMD in RPD eyes^17^. In this study, thinner SFCT, a rapid decrease in SFCT, a larger RPD area at baseline, a rapid increase in the RPD area, and the presence of late AMD on fellow eye were significant risk factors for late AMD in RPD eyes. Further, those with larger RPD areas (≥ 16 segments) at the first visit showed a higher cumulative probability of late AMD development than did those with smaller RPD areas (< 16 segments). In another Asian study (Japan)^18^, 45% of RPD eyes developed late AMD during a 60-month follow-up, which is higher than the results of this study. Although the patients in both studies were Asian, there are several differences, such as a younger mean age (67 years vs. 79 years), a lower proportion of males (7% vs. 49%), and a smaller number of eyes with large drusen (30% vs. 64%) in the present study.

Previous studies demonstrated that confluent and ribbon types increased the risk of late AMD^2,18^. However, only the RPD area was investigated in this study, and there was no comparison analysis between RPD area and RPD distribution pattern for the risk of late AMD. Future studies investigating both RPD area and RPD distribution pattern, will be needed to investigate reveal the comparative risk relationship of these factors for late AMD.

The limitations of this study included the small number of patients, recruited from a single center, and the retrospective study design. There are other limitations as well. First, the RPD area measurement was performed using a semi-quantitative UWF imaging method. The individual segments in the concentric ring method do not have the same area size. Thus, subtle RPD changes and regional variations within a segment might not be detected in this study. Furthermore, the foveal area was not included in this method because of the difficulties in detecting subtle RPD changes in the foveal area. Second, UWF imaging might be not the gold standard for detecting RPD outside the arcade, and the RPD area could be underestimated because it is a pseudo-color photography. However, it showed acceptable detectability, reliability, and reproducibility in previous studies^19,20^. Further studies with a quantitative area measurement method and more accurate imaging devices are needed to confirm the results of this study.

In conclusion, the RPD areas progressively increased over time in all eyes with RPD. A large size and rapid increase in the RPD area appeared to be risk factors for late AMD development. Thus, assessment of the RPD area should be included in the routine examination of RPD eyes.

## Methods

This study was conducted in accordance with the Declaration of Helsinki and was approved by the institutional review board of the Samsung Medical Center in Korea. Given the retrospective nature of the study and the use of anonymized data, requirements for informed consent were waived by the institutional review board.

### Patient selection

**Figure 4:**
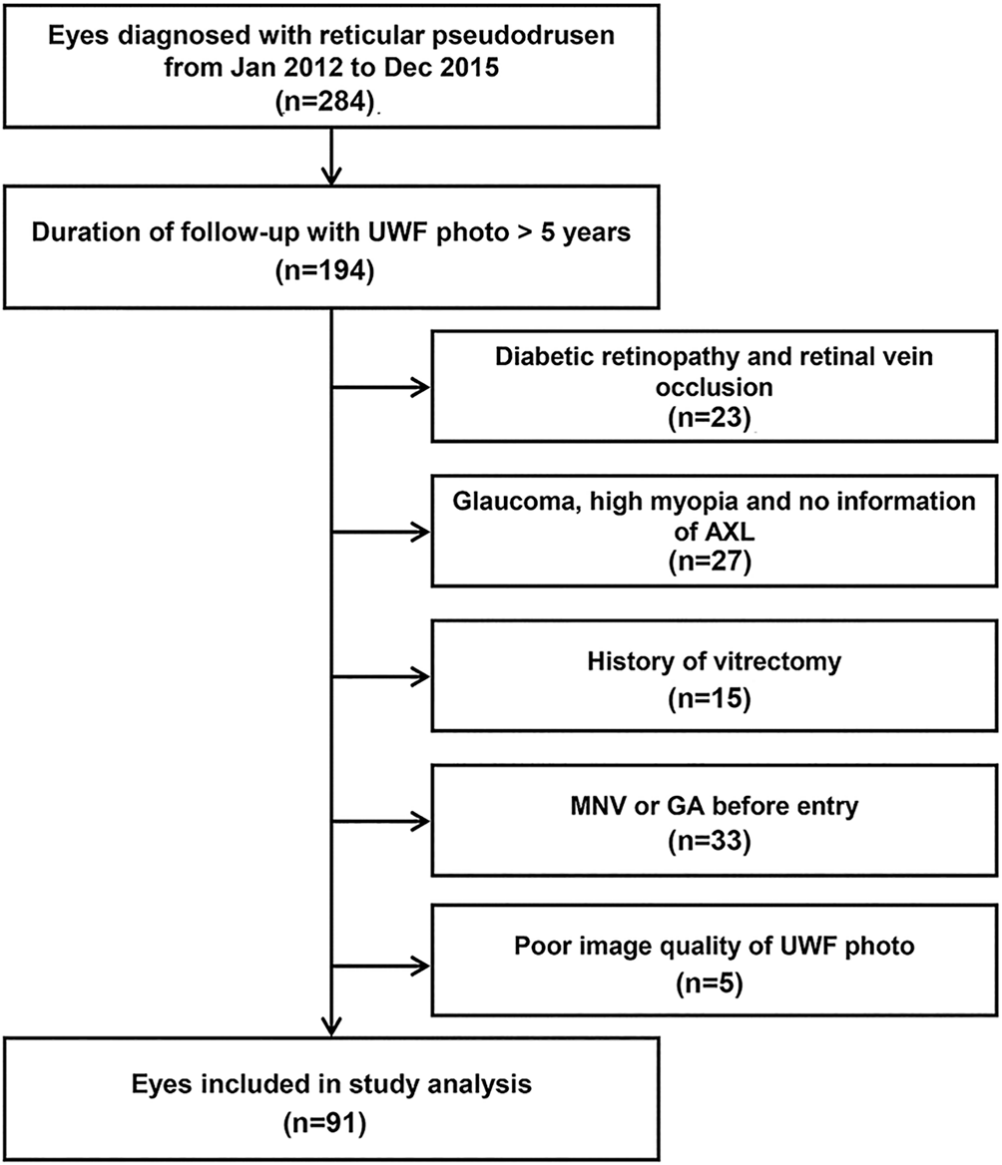
Flowchart demonstrating the selection process for the eyes included in the study.

We retrospectively analyzed the medical records of patients diagnosed with RPD at the Samsung Medical Center between January 2012 and September 2015. Patients were included if they were examined repeatedly over a 5-year follow-up period, using UWF imaging. Exclusion criteria were as follows: any ophthalmologic conditions that could affect the diagnosis of RPD or analysis of the area affected with RPD (e.g., any laser scars, retinal detachment, diabetic retinopathy, hypertensive retinopathy, central serous chorioretinopathy, retinal vein occlusion, and severe epiretinal membrane), any signs or history of MNV and GA in the study eye at baseline; history of vitrectomy before entry or during the study period; glaucoma; high myopia (axial length ≥ 26.5 mm or spherical equivalent ≥ 6 diopters), incomplete examinations, poor image quality of UWF photographs, and loss to follow-up. Figure 4 shows a flowchart of the selection process for the eyes included in the study.

### Ocular examination and image acquisition

All patients underwent a comprehensive ophthalmic examination, including measurements of BCVA, refractive error, slit-lamp biomicroscopy, fundus examination, and UWF photography (Optos 200Tx, Optos PLC, UK). Patients also underwent multimodal imaging, including color fundus photography (CFP; TRC 50 IX, Topcon, Japan), red-free photography, near-infrared reflectance (NIR), fundus autofluorescence (FAF), and optical coherence tomography (Spectralis HRA + OCT, Heidelberg Engineering, Germany or DRI OCT Triton, Topcon, Japan). Fluorescein angiography (FA) and indocyanine green angiography (ICGA; Spectralis HRA + OCT or Optos 200Tx) were also performed.

### Diagnosis of reticular pseudodrusen and age-related macular degeneration

For the diagnostic criteria of RPD, the same criteria as those of the previous report were used^17^.

AMD was classified, according to the Clinical Classification System of the Beckman Initiative for Macular Research Classification Committee^21^. The definition and classification of MNV followed the criteria proposed by the CONAN (Concensus on Neovascular Age-related macular degeneration Nomenclature) study group^22^. GA was defined as a sharply demarcated hypopigmented area with visible large choroidal vessels in CFP and hypoautofluorescent in FAF, with a diameter of at least 175 μm^23,24^.

### Analysis of ultrawide-field and optical coherence tomography images

**Figure 5:**
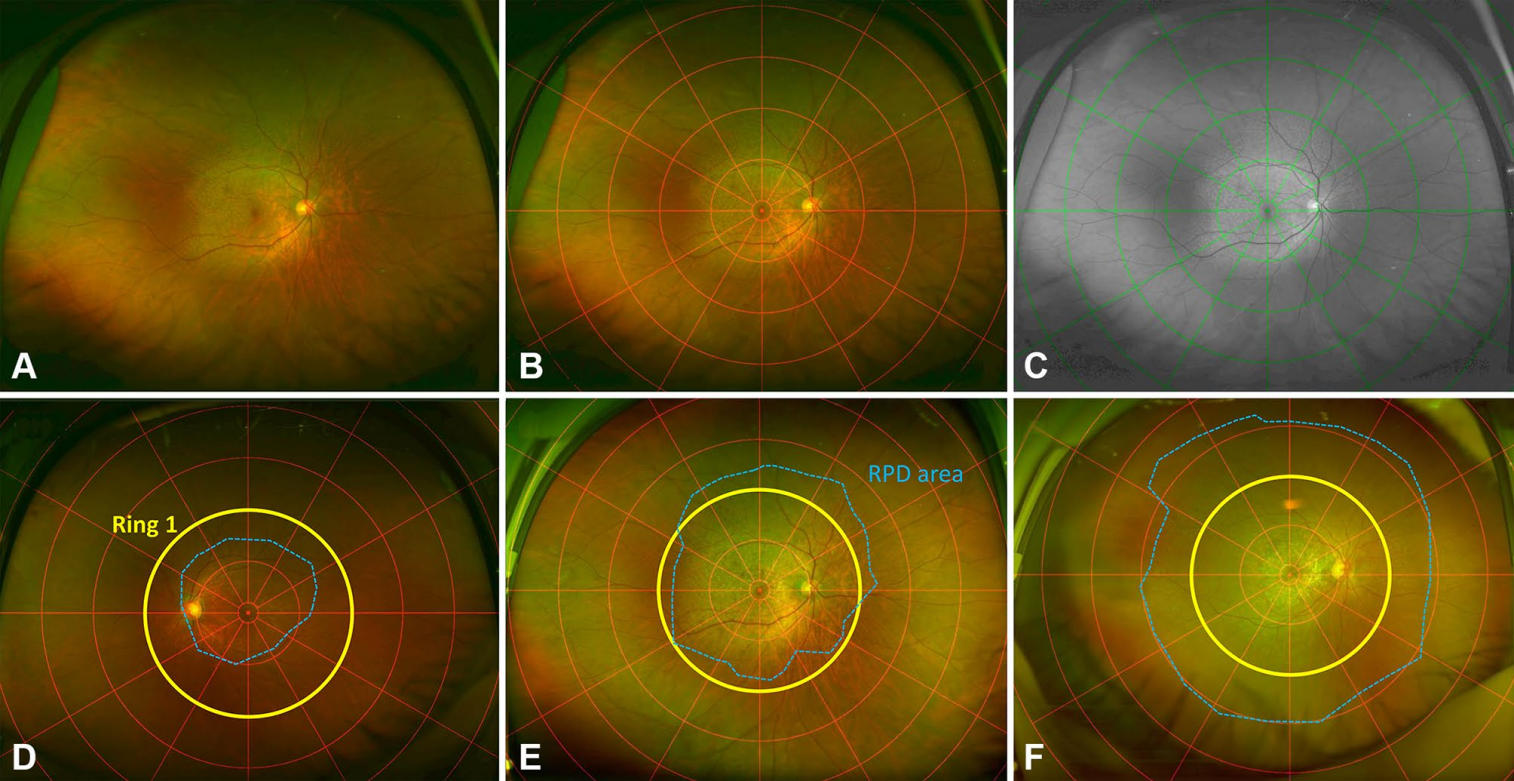
Analysis of fundus distributional area and type of reticular pseudodrusen (RPD). (**A**) Ultrawide-field photograph of the eye of a 50-year-old woman. The image of concentric rings superimposed on (**B**) the UWF pseudocolor photograph and (**C**) red-free photograph. After RPD area (blue dotted line) had been identified, the number of affected segments, excepting the small central circular area (the fovea), was counted. Fundus distributional types were classified into (**D**) central type, (**E**) intermediate type, and (**F**) extensive type. The central type was defined as RPD present within Ring 1 (yellow ring). The intermediate type was defined as RPD present beyond Ring 1 in one to three quadrants. The extensive type was defined as RPD present beyond Ring 1 in all quadrants.

The RPD area in UWF imaging was semi-quantitatively analyzed using the concentric rings method, as indicated elsewhere^25^. After the concentric rings were superimposed onto each of the selected UWF images using Adobe Photoshop 2020 (Adobe Inc., San Jose, CA, USA), the number of segments in which RPD occurred was counted (Fig. 5A–C). For analyzing UWF images, we used both of UWF pseudocolor images (Fig. 5B) and UWF red-free images (Fig. 5C). UWF red-free images were made from UWF pseudocolor images using green channel in Adobe Photoshop 2020^20,26^. For the analysis, UWF photographs taken at the first visit, the time of late AMD diagnosis, and the final visit were used. The area within the first ring (the foveal area) was not included in the analysis. The macula was defined as the area consisting of segments within the M ring (the second ring).

The fundus distributional type of RPD was determined according to the extent of the affected retinal area. Eyes were divided into three types: central, intermediate, and extensive. The central type was defined as one where RPD was present within Ring 1 (the third ring). The intermediate type was defined as one where RPD was present beyond Ring 1 in one to three quadrants. The extensive type was defined as one where RPD was present beyond Ring 1 in all four quadrants (Fig. 5D–F). All UWF images were assessed by two investigators (J.M.Y and Y.J.C.), and in case of disagreement, a senior interpreter (D.I.H.) made the final decision.

CRT at the time of the first visit and the final visit were obtained from the thickness map profile of a SD-OCT volume scan. Automated segmentation (internal limiting membrane and Bruch’s membrane) was evaluated, and errors were manually corrected prior to CRT measurement.

SFCT was defined as choroidal thickness at the fovea. The SFCT was manually measured using a horizontal OCT scan image. A single examiner (J.M.Y.), who was blinded to the patient’s identity and timing of the OCT examination, performed all measurements.

### Statistical analysis

Changes in characteristics between the first visit and the final visit in the entire cohort were analyzed using the generalized estimating equation method adjusted by interval year. Differences in distribution or changes in the RPD area among each retinal area were analyzed using the Wilcoxon signed ranks test, paired *t* test, and one-way analysis of variance. Moreover, Pearson’s correlation analysis was performed to investigate the associations among parameters.

Intergrader agreement was assessed using the intraclass correlation coefficient for continuous variables and Cohen’s kappa coefficient for categorical variables.

The Contal and O’Quigley method was used to determine a cut-off value for classifying eyes into two groups, as distinct from 3 classifications above, according to the RPD area at the first visit; the calculated cut-off value was 16 segments^27^. Kaplan‒Meier analysis and the log-rank test were used to compare survival experiences (time-to-progression) between the two groups. The hazard ratios (HRs) for the associations between potential risk factors for late AMD were determined using the Cox proportional hazards model. Factors with p < 0.05 on univariate analysis and previously reported risk factors of late AMD (SFCT, large drusen of which size is greater than or equal to 125 μm, pigmentary changes, and late AMD on fellow eye)^17,28^ were included in the multivariate Cox proportional hazards model. Adjusted HRs with 95% confidence intervals (CIs) were calculated. Statistical analyses were performed using IBM SPSS software (version 27.0; IBM, Armonk, NY, USA), SAS (version 9.4; SAS Institute Inc., Cary, NC, USA), and R (version 4.0.3; Vienna, Austria; http://www.R-project.org/). A p value of less than 0.05 was considered significant.

## Data Availability

The datasets generated during and/or analysed during the current study are available from the corresponding author on reasonable request.
